# Facile Tailoring of Contact Layer Characteristics of the Triboelectric Nanogenerator Based on Portable Imprinting Device

**DOI:** 10.3390/ma13040872

**Published:** 2020-02-15

**Authors:** Sumin Cho, Sunmin Jang, Moonwoo La, Yeongcheol Yun, Taekyung Yu, Sung Jea Park, Dongwhi Choi

**Affiliations:** 1 Department of Mechanical Engineering, Kyung Hee University, 1732 Deogyeong-daero, Yongin, Gyeonggi 17104, Korea; tnals1404@khu.ac.kr (S.C.); jsm2962@khu.ac.kr (S.J.); etm024@khu.ac.kr (Y.Y.); 2 School of Mechanical Engineering, Korea University of Technology and Education (KOREATECH), Cheonan, Chungnam 31253, Korea; mla@koreatech.ac.kr; 3 Department of Chemical Engineering, Kyung Hee University, 1732 Deogyeong-daero, Yongin, Gyeonggi 17104, Korea; tkyu@khu.ac.kr

**Keywords:** Triboelectric nanogenerator, energy harvesting, contact layer, biomechanical energy

## Abstract

Renewable energy harvesting technologies have been actively studied in recent years for replacing rapidly depleting energies, such as coal and oil energy. Among these technologies, the triboelectric nanogenerator (TENG), which is operated by contact-electrification, is attracting close attention due to its high accessibility, light weight, high shape adaptability, and broad applications. The characteristics of the contact layer, where contact electrification phenomenon occurs, should be tailored to enhance the electrical output performance of TENG. In this study, a portable imprinting device is developed to fabricate TENG in one step by easily tailoring the characteristics of the polydimethylsiloxane (PDMS) contact layer, such as thickness and morphology of the surface structure. These characteristics are critical to determine the electrical output performance. All parts of the proposed device are 3D printed with high-strength polylactic acid. Thus, it has lightweight and easy customizable characteristics, which make the designed system portable. Furthermore, the finger tapping-driven TENG of tailored PDMS contact layer with microstructures is fabricated and easily generates 350 V of output voltage and 30 μA of output current with a simple finger tapping motion-related biomechanical energy.

## 1. Introduction

Many organizations have encouraged studies on energy harvesting technology, which converts various forms of renewable energies into electrical energy, due to the rapid depletion of fossil fuels, such as oil, coal, and natural gas [1]. Among various energy harvesters, the triboelectric nanogenerator (TENG), which generates electricity through coupling of triboelectrification between two different materials and resultant electric al induction, is attracting close attention in harvesting numerous abandoned energies, such as biomechanical energy, wind energy, and vibration energy [2,3,4,5,6,7,8,9,10,11,12,13,14,15,16,17,18]. The advantageous characteristics of TENG, such as high shape adaptability and accessibility and easy processability, make it a promising functional biomechanical energy harvester [5,6,7,13]. For example, TENG can be easily constructed and realized using biocompatible materials without harmful materials. Such biocompatible TENG can be injected or inserted in the human body to harvest various types of human-related biomovements, such as heart beating, joint movement, eye blinking, and footstep. Triboelectricity is a spontaneous electrification phenomenon that occurs due to sequential contact of two different material surfaces, which are often called contact layers. Electrical charge pairs are formed on their surface as a result of triboelectricity between two contact layers. Here, the sign and amount of the electrical charges are determined by the electron affinity or work function difference between the materials of two contact layers. Following separation of two contact layers breaks electrical charge pairs on contact layers, and resultant formation of net electrical charges generate an electric field around them. Then, electrical induction is caused in the conductive layer near the contact layer, and this spontaneous electrical induction can be considered a movement of electrical charges resulting in generation of electric current. The above mentioned simple operation mechanism makes TENG a promising energy harvester [2,3,4,5,6,7,8,9,10,11,12,13,14,15,16,17,18,19,20,21,22,23,24,25,26,27,28,29,30]. In addition, cost-effectiveness, simple fabrication with material selection diversity, high shape adaptability, and high accessibility are emphasized as the other advantageous features of TENG [19,20,21,22,23,24]. Since the first proposal of TENG, much effort has been made to increase its electrical output performance by using various strategies [25,26,27,28,29,30]. The fundamental operation mechanism of TENG is based on repetitive contact and separation of contact layers. Thus, the easiest and straightforward way to control the electrical output performance of TENG is to tailor its contact layer. A frequently utilized method to increase the electrical output performance of TENG is to insert micro/nanostructures on the surface of the contact layer. According to literature, inserting micro/nanostructures increases local contact pressure and local contact area; this condition generates a large amount of electrical charges on the contact layer of TENG [5,31,32,33,34,35,36,37]. Previous methods used to fabricate micro/nanostructures on the contact layer of TENG are based mostly on two approaches: Surface etching and replication. The former refers to selective polymer surface etching with the help of reactive ions to increase surface roughness. Although this approach successfully enhances the electrical output performance of TENG, the requirement of high-vacuum and high-temperature processes impairs the accessibility of the method. In addition, the shape and dimensions of the structures cannot be easily controlled using this method. On the contrary, surface replication refers to the method of imprinting the surface and is regarded as a powerful technique to introduce desired patterns on the surface of the TENG contact layer. In general, the liquid-phase polymer is poured into a mold with micro/nanocavities, and the solidification process follows to fabricate the surfaces with structures. The surface replication method is often considered a fabrication process one step closer to mass production, which is essential for further commercialization, due to the reusability of the mold and relatively low manufacturing costs. In addition, the equipment accompanied with surface replication process has a high potential to become portable because it does not generally require extreme processing conditions, such as high temperature and vacuum. The fabrication technology always plays a crucial role in new research concepts. Thus, surface replication for TENG fabrication should be intensively studied for the future practical utilization of TENG.

In this study, we develop a portable imprinting device to fabricate TENG in one step by easily tailoring the characteristics of its polydimethylsiloxane (PDMS) contact layer, such as thickness and morphology of the surface structures. These characteristics are critical to determine the electrical output performance of TENG. PDMS, which belongs to a group of polymeric organosilicon compounds, is widely utilized as a contact layer of TENG and has various advantageous characteristics of easy processibility, optical clearness, chemical inertness, and biocompatibleness [38,39,40,41,42,43,44,45,46,47,48,49]. Adoption of the rationally designed screw enables us to elaborately and uniformly control the thickness of the PDMS contact layer. All parts of the device are 3D printed with high-strength polylactic acid (PLA). As a result, the device has a lightweight characteristic, which is favorable for being portable. Conventionally available sandpapers and a wrapping film are utilized as molds for the structured PDMS contact layer with various thicknesses given that the device has no limitation on mold selection. Furthermore, by using the developed PDMS contact layer with microstructures, the finger tapping-driven TENG (FT-TENG) is fabricated to harvest finger tapping motion-related biomechanical energy.

## 2. Materials and Methods

### 2.1. Development and Operation of the Portable Imprinting Device

All parts of the portable imprinting device were rationally designed and 3D printed with high-strength PLA. To replicate microstructures on the surface of the PDMS contact layer, 150 grits, 320 grits sandpapers, and a conventionally available wrapping film were utilized as molds. The sandpaper and the wrapping film were attached to the mold-attached plate, and the thickness of the PDMS contact layer was controlled. To control the thickness of the PDMS contact layer, PDMS was initially filled between the bottom part of the device housing and the contact part; then, the thickness was set by adjusting the position of the screw. Finally, the portable imprinting device was placed into an oven for 2 h to cure the PDMS after vacuuming.

### 2.2. Characterization of the PDMS Contact Layer

Optical microscope (VHX-950F, KEYENCE, Osaka, Japan) was utilized to observe the resultant surface structure of the PDMS contact layer. The magnification of the microscope was set to ×200. In addition, the height of the surface structure was measured using a 3D stereoscopic system. Contact angle was measured using smart drop (SDS-TEZD10013, FEMTOFAB, Seongnam, Korea). 

### 2.3. Measurement of the Electrical Output Performance of the Patterned PDMS by Using TENG

A vertical contact-separation mode was adopted to measure the output performance of the TENG. The vibration machine (KD-9363ED-41E, Kingdesign, New Taipei City, Taiwan) was used to create vertical vibration. Output voltage (V_O_) was measured by directly connecting the oscilloscope (DS1074z, Rigol, Beaverton, OR, USA) equipped with the high-voltage probe (DP–50, Pintek, New Taipei City, Taiwan) to the TENG. The internal resistances of the oscilloscope and the high-voltage probe were 1 and 15 MΩ, respectively. The output current (I_O_) was measured by connecting the current amplifier (SR-570, Stanford Research Systems, Sunnyville, CA, USA) and oscilloscope to the TENG.

## 3. Results and Discussion

### 3.1. Operation Mechanism of the Portable Imprinting Device 

**Figure 1:**
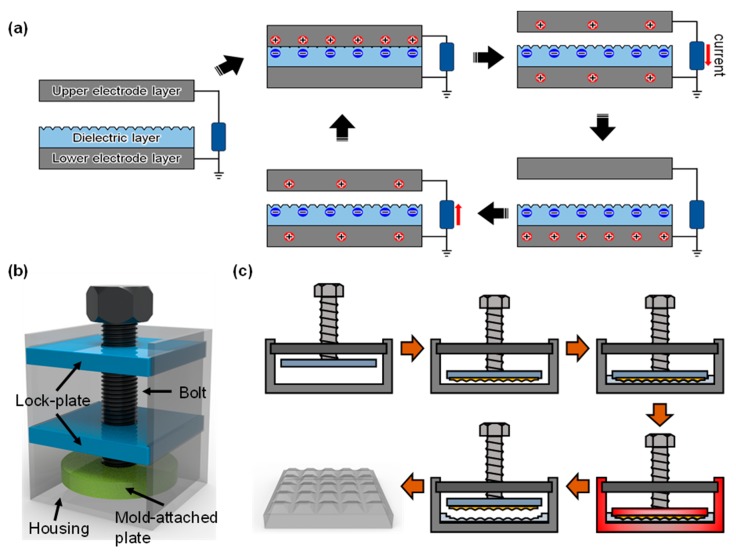
(**a**) Operation mechanism of the triboelectric nanogenerator (TENG). (**b**) Schematic of the developed imprinting device. (**c**) Schematic of the operation mechanism of tailoring the contact layer of the TENG.

The basic operating mechanism of the popular TENG with the vertical contact-separation mode is briefly shown in Figure 1a. The TENG is composed of the dielectric contact layer, the lower electrode layer, and the upper electrode layer, which contact the dielectric contact layer during its operation. Here, the dielectric contact layer plays a critical role in generating electrical charge. When the upper electrode layer contacts the dielectric layer, electrical charge pairs are generated on the contact interface. The sign and amount of the electrical charges are highly dependent on the characteristics of the dielectric contact layer. Notably, the generation of charges follows the electron affinity difference between the upper electrode layer and the dielectric contact layer. Here, the positive (negative) electrical charges are assumed to be generated on the surface of the upper electrode layer (dielectric layer). As the separation distance between the upper electrode layer and the dielectric layer becomes large after their contact, the separated electrical charges generate an electric field, which repulses the electrical charges with the same sign. During this separation process, the electrical current flows because electrons in the lower electrode layer move to the upper electrode layer to satisfy electro-neutrality. After perfect receding of the upper electrode layer from the dielectric layer, the upper electrode layer becomes electrically neutral, while the lower electrode layer is positively charged due to lack of electrons. As the upper electrode layer and the dielectric layer becomes close again, the electrons in the upper electrode layer tend to move back to the lower electrode as a response to the electric field. The TENG can generate an alternating current by repeating the contact and separation processes, and this condition generates electricity. In accordance with the above-mentioned electricity generation mechanism of the TENG, the two important aspects to determine its electrical output performance are (1) the amount of the generated electrical charges on the surface of the dielectric layer and (2) the resultant electric field to induce the electrical charges on the nearby electrode layer. The most straightforward approach to control such aspects is to tailor the characteristics of the dielectric contact layer, such as topography and thickness. Since the first proposal of TENG in 2012, the importance of tailoring the contact layer properties has been continuously emphasized by researchers to enhance its electrical output performance. On this basis, we develop a portable imprinting device to easily tailor the characteristics of the PDMS contact layer of TENG, such as thickness and morphology of the surface structures, as shown in Figure 1a. According to the literature, PDMS is widely utilized as a contact layer material of TENG and has various advantageous characteristics of easy processibility with mild curing condition, optical clearness, chemical inertness, and biocompatibility [38,39,40,41,42,43,44,45,46,47,48,49]. In addition, the PDMS-based TENG can generate relatively high electrical output performance [40]. The crucial part of the device to precisely control the thickness of film is the mold-attached plate with the screw, which can change the rotational motion to delicate linear motion. The mold-attached plate replicates the surface of the mold for easy fabrication of surface structures. The mold-attached plate is designed to be sufficiently thick to endure thermal deformation during the PDMS curing process. In addition, two lock-plates are utilized to guide the linear screw movement during its rotation; aligning the position of the screw is difficult with only one lock-plate due to insufficient fixation. The fabrication process of the PDMS contact layer by using the proposed imprinting device is shown in Figure 1c. Initially, the mold, which possesses the negative relief of the desired surface structure, is attached on the mold-attached plate, and the thickness of the contact layer can be minutely adjusted by rotating the screw. Then, the mixture of the PDMS with curing agent is poured to fill the space between the housing and the mold. The entire device is placed into a vacuum chamber to remove air bubbles. The simple configuration of the imprinting device with lightweight characteristic makes the device portable. Thus, the whole device can be freely moved. After the vacuum process, the whole device is placed into the hot oven for a sufficient time to fully cure the PDMS. In this process, the curing temperature should be controlled under 210 °C to prevent melting of the device. Then, the PDMS contact layer with desired thickness and surface structures can be easily fabricated by detaching it from the device.

### 3.2. Tailoring the Characteristics of the PDMS Contact Layer

**Figure 2:**
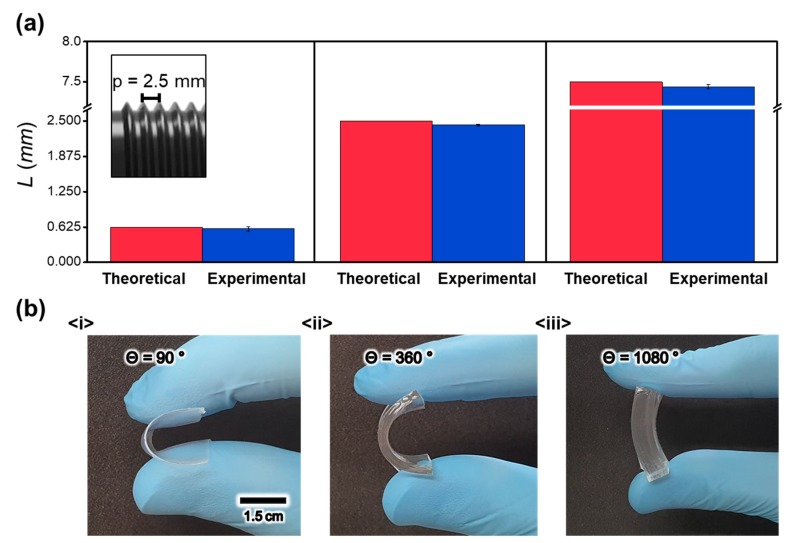
(**a**) Comparison between theoretical and experimental film thicknesses; (inset) pitch of the screw. (**b**) Thickness of the fabricated film depending on θ.

The developed imprinting device enables us to easily control (1) the thickness and (2) the morphology of the PDMS contact layer. To investigate its controllability, the thickness control experiment is conducted. The resultant thickness of the PDMS contact layer can be theoretically calculated. Thus, the thickness of the PDMS contact layer, which is fabricated using the imprinting device without the mold, is compared with its theoretical value. Here, the linear distance (L) of the screw movement determines the thickness of the PDMS contact layer. In general, L as a response of the screw rotation can be calculated as follows:

(1) $$L=p\;\times \;\frac{\theta }{360{}^{\circ}},$$

where p and θ represent the pitch of the screw and the angle of rotation, respectively. is set to 2.5 mm, as shown in Figure 2a. Thus, a full rotation of the screw results in a linear movement of 2.5 mm. According to Equation (1), the theoretical thicknesses of the PDMS contact layer become 0.625, 2.5, and 7.5 mm when the values of are changed to 90, 360 (one full revolution), and 1080°, respectively. The thickness of the fabricated film is characterized using the five-point (top, bottom, right, left, and center) probe measurement method. As plotted in Figure 2b, the theoretical thickness of the PDMS contact layer in all cases is slightly (around 50 μm) larger than the experimentally measured thickness. Given that the difference does not increase as increases, this slight difference can be considered due to the initial misalignment, which can be further removed using the calibration procedure; the thickness is well controlled using the proposed imprinting device. Figure 2b shows the image of the fabricated PDMS contact layer with various thicknesses.

**Figure 3:**
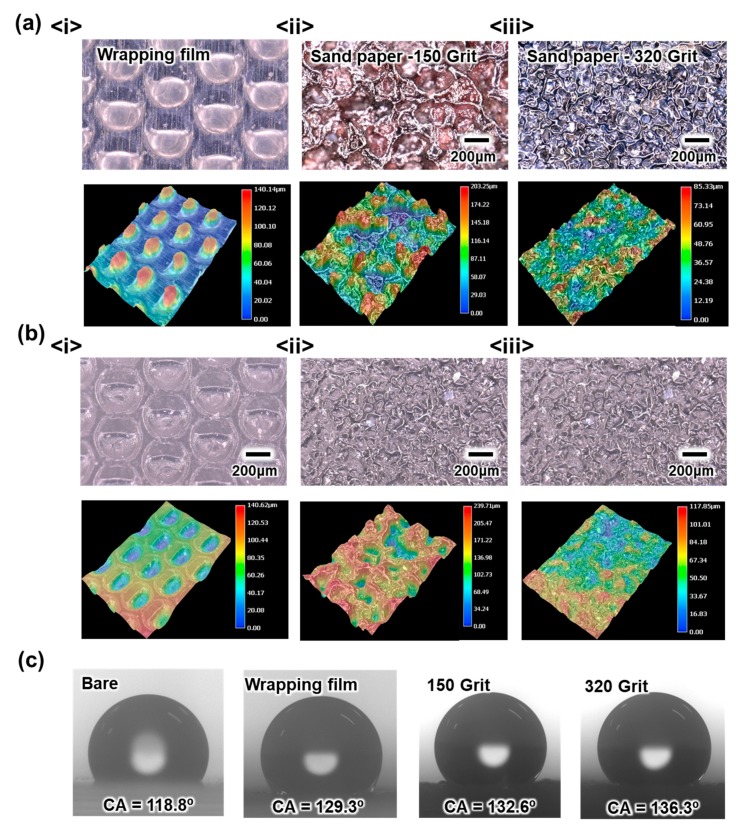
(**a**) Two-dimensional (2D) and three-dimensional (3D) observation result of surface structures on the mold. (**b**) 2D and 3D observation result of the surface structures on the fabricated film. (**c**) The static contact angle (CA) of the polydimethylsiloxane (PDMS) film with different surface structures.

The important role of the present imprinting device other than controlling the thickness of the contact layer is replication of the surface, which enables the structures on its surface to fabricate. The surface replication process of the present imprinting device does not require an extreme processing condition, such as high temperature and pressure. Thus, nearly no limitation is imposed on the selection of mold to be utilized. Accordingly, conventionally available films, such as a wrapping film and sandpaper with various grits, are utilized as molds for surface replication by using the present device, as shown in Figure 3. Figure 3a,b show the 3D microscopic images of the molds and the replicated PDMS surfaces, respectively. In the case of the wrapping film, the presence of the well-aligned structures on its surface enables to quantitatively analyze the surface replication quality. The successful surface replication by using the present imprinting device can be confirmed by comparing the heights of the structures on the wrapping film and on the replicated PDMS surface. The presence of the structures on the surface of the contact layer can be further indirectly confirmed by measuring the static effective contact angle ($\theta_{e}$) of the water droplet on the surface. According to the wetting equation, which describes the effective contact angle (CA) for a liquid on the heterogeneous surface, the presence of the structures on the hydrophobic surface strengthens its hydrophobicity; thus, $\theta_{e}$ increases, as follows:

(2) $$\cos\theta_{e}=r\cos\theta_{c},$$

where r and $\theta_{c}$ are the roughness determined by the presence of the surface structures and the original static contact angle of the surface without structures, respectively. The enhancement of the hydrophobicity of the surface, as described in Figure 1c, confirms the replication of the structural surface with the proposed imprinting device.

### 3.3. Electrical Output Performance of TENG with Structured PDMS Contact Layer

**Figure 4:**
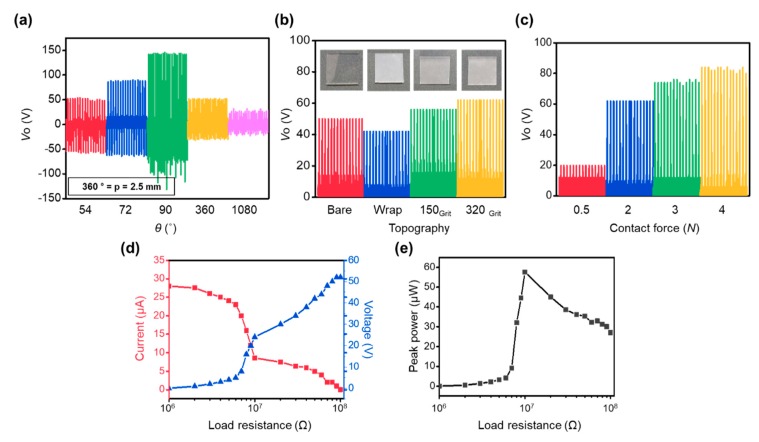
Output voltage comparison under various parameters such as (**a**) thickness of the film depending on θ, (**b**) type of the mold, and (**c**) applied contact force to the TENG. (**d**) Output voltage, current, and (**e**) output power under different load resistances.

The TENG with the fabricated PDMS contact layer is developed by simply placing the aluminum (Al) layer on the bottom of the housing of the imprinting device, as shown in Figure 1b, to investigate the effect of the thickness and the surface structure of the PDMS contact layer of the TENG fabricated by the proposed imprinting device on the electrical output performance. Then, exactly the same procedure to tailor the characteristics of the PDMS contact layer is conducted, and the TENG with the PDMS contact layer can be fabricated in one step due to the presence of the bottom electrode layer. TENGs with various thicknesses are fabricated by varying θ, and the output voltages (*V*_O_s) during their operation with the vertical contact-separation mode described in Figure 1a with the Al upper electrode layer are measured using an oscilloscope, as shown in Figure 4a. The plot shows the bell-shaped behavior as the thickness of the PDMS contact layer increases. When the thickness of the PDMS contact layer increases from θ = 54° (thickness = 0.375 mm) to θ = 90° (thickness = 0.625 mm), *V*_O_ increases. According to the literature, this increased behavior of *V*_O_ with increased thickness of the contact layer could be conjectured due to the increased effect of the total amount of the charges in the PDMS contact layer [50]. Although there is a difference in the range of the thickness of the contact layer, the previous literature with numerical simulation has shown that there would be a positive effect of the thick contact layer on the output performance [50]. As thickness further increases from θ = 90°, the TENG with the thick contact layer results in low *V*_O_ due to the effect of the weakened electric field. When the thickness of the PDMS contact layer increases, the distance between the generated charges on its surface and the lower electrode layer increases, which weakens the electric field. The weak electric field induces a small amount of the electrical charges on the bottom electrode layer during operation of the TENG. Thus, the too thick contact layer is not preferred to generate electrical output performance. The result shown in Figure 4a directly shows that the thickness of the PDMS contact layer, which significantly affects the output performance of the TENG, can be easily controlled and even optimized without any cumbersome process by using the present imprinting device. 

In addition to the thickness of the contact layer, the effect of the topography on the surface of the PDMS contact layer is investigated. The TENG with the PDMS contact layer (θ = 90°) is utilized to investigate the effect, as shown in Figure 4b. In the case of the following experimental results, for its intuitive comparison in the plot, the only positive peaks of *V*_O_ would be utilized. Comparing *V*_O_s generated from the TENG with various surface structures indicates that the TENGs with sandpaper replicated structures generate approximately 1.5 times larger *V*_O_s than that generated from the TENG without any structures (bare). The result corresponds with those of numerous previous studies, which have shown the positive effect of the presence of surface structures on the electrical output performance of TENG. According to these studies, the formation of the surface structures significantly increases the local contact pressure under the same applied contact force. This condition further increases the contact area between two contact surfaces. As a result, a high amount of electrical charges is generated on the contact surface. However, the TENG with the replicated structures from the wrapping film generates lower *V*_O_ than that generated from bare. Although additional investigation of this interesting phenomenon is required, it can be conjectured due to the reduced contact area. As shown in Figure 3, the replicated surface structure by using the wrapping film is intaglio, and this well-aligned negative relief on the surface of the contact layer can reduce the effective contact area. Thus, less electrical charges can be formed during operation of the TENG. In other words, the formation of the surface structures on the contact layer does not always enhance the electrical output performance. For further analyses on the effect of the surface topography, we can apply the concept of flexoelectricity, which is a property of a dielectric material whereby it exhibits an electrical polarization induced by a strain gradient on the surface [51]. According to the recent literature, the output voltage generated from the TENG would be inversely proportional to the one third of the radius of surface structures on the contact layer, when we apply the force to the contact layer with the well-defined spherical cap structured contact layer [51]. To apply such an idea into our experimental result, we newly calculate the average roughness (S_a_) of the replicated PDMS contact layer with 150 and 320 grits sandpaper as 1.48 and 0.79 µm, respectively. As an arithmetical mean deviation of the assessed surface profile, S_a_ in this study is utilized to the abovementioned idea instead of radius of the surface structure on the contact layer. According to the literature, the ratio of *V*_O_ between two cases by using the S_a_ would be 0.82. In the experimental data in Figure 4b, the ratio of *V*_O_ is calculated as 0.9. Although there is a slight difference between two values, the analyses indirectly show that the flexoelectricity driven by the surface topography could be the origin of triboelectricity. The slight difference may arise from the utilization of S_a_ instead of radius of the surface structure and thus, the future work with the well-defined surface structures would enable us to further investigate the effect.

Furthermore, the effect of the contact force on *V*_O_ is investigated using the TENG with the PDMS contact layer (θ = 90° with 320 grits sandpaper). As shown in Figure 4c, the high contact force applied to the TENG enhances the electrical output performance. According to previous studies, the increase in contact force generates more electrical charges on the surface of the TENG contact layer. This condition enhances the electrical output performance of TENG, which corresponds to the result shown in Figure 4c [52,53,54]. Another electrical characteristic of TENG to be investigated is the maximum power transfer depending on load resistance. In general, the energy harvester plays a role to supply power to the electronic device, and the energy harvesting performance can vary with the amount of load resistance, where the generated power is transferred. Thus, the generation of peak power from the TENG depending on the external load resistance is investigated. As shown in Figure 4d,e, the power at the external load resistance is maximized to 60 μW when the resistance reaches 10 MΩ. This finding indicates that the present TENG has an internal resistance of 10 MΩ. This experimental result can be utilized in the future for impedance matching to maximize the performance of TENG. In addition, if the TENG with further increased output performance is required, we could consider applying the UV/plasma treatment as a post-process (see Figure S1). According to the literatures, post-treatment of the PDMS contact surface to modify the surface chemistry with application of the UV beam can enhance the output performance of the TENG and thus, if we can integrate such post-treatment into our imprinting scheme, that would help further increase the output performance of the TENG [55,56]. 

### 3.4. FT-TENG Fabrication and Application

**Figure 5:**
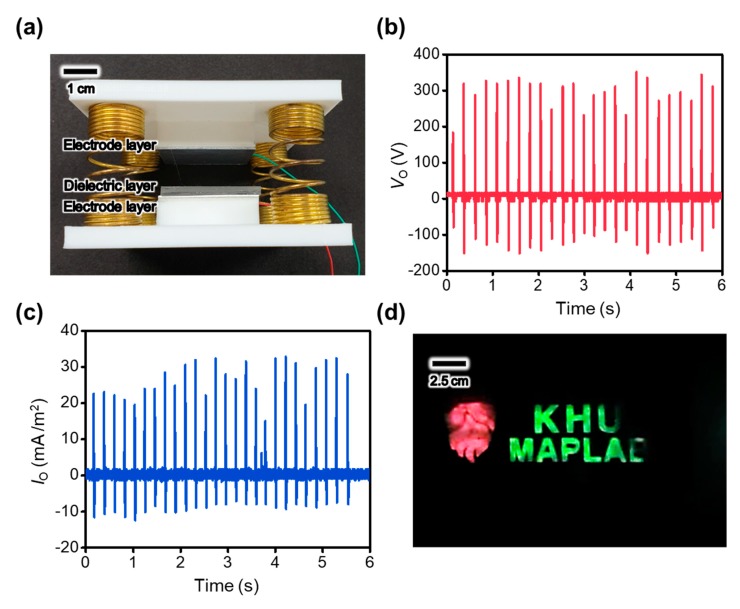
(**a**) Image of the finger tapping-driven TENG (FT-TENG) (9 cm^2^ in contact area) to harvest the finger tapping motion related biomechanical energy. (**b**) Output voltage and (**c**) output current generated by the FT-TENG with simple finer tapping motion. (**d**) Custom-built light emitting diode (LED) display lighting by using the FT-TENG.

The FT-TENG is developed, as shown in Figure 5a, on the basis of the PDMS contact layer with the highest electrical output performance. The structured PDMS contact layer attached on the Al layer and the other Al layer are utilized as two separate components of the FT-TENG, which contact and separate each other to generate electricity. To facilitate the operation of FT-TENG, the two components are attached and supported by the PLA plates, which are elaborately designed and 3D printed. In addition, four compressive springs located at the vertices of the plates maintain the position of the components with a separated status through spring resilience. Applying the force to the upper PLA plates of the FT-TENG by using the single finger pressing motion generates 350 V of *V*_O_ and 30 μA of the output current, as shown in Figure 5b,c, respectively. The output performance of the FT-TENG is sufficient to light the custom-built light-emitting diode display, as shown in Figure 5d. This proof of concept demonstration of the operation of the FT-TENG to harvest the finger pressing motion shows that the present FT-TENG can generate electricity from human body motion to power portable electronic devices.

## 4. Conclusions

A portable imprinting device is developed to tailor the characteristics of the contact layer of TENG. The thickness of the contact layer can be elaborately controlled by rotating the screw bolt, and the surface structure on the contact layer can be easily inserted on the basis of the replication process with cost-effective characteristics. The tailoring of the characteristics of the TENG contact layer enhances the electrical output performance. Furthermore, the FT-TENG of tailored PDMS contact layer with microstructures is fabricated and easily generates 350 V of output voltage and 30 μA of output current by using the simple finger tapping motion-related biomechanical energy. The developed imprinting device and the proposed facile method to tailor the characteristics of the contact layer of the TENG in this study will provide an approach to intensively study the effect of the contact layer of the TENG on its electrical output performance for its future utilization as an effective biomechanical energy harvester.

## Supplementary Materials

The following are available online at https://www.mdpi.com/1996-1944/13/4/872/s1. Figure S1: Output voltages generated from the TENG with the pristine PDMS contact layer and the ozone treated PDMS contact layer.
