# Supplementary material for: Facile Tailoring of Contact Layer Characteristics of the Triboelectric Nanogenerator Based on Portable Imprinting Device
Source: Materials (Basel). 2020 Feb 15;13(4):872. doi: 10.3390/ma13040872 (PMC7079606; doi:10.3390/ma13040872)
Supplement: Supplementary file 1 [file materials-13-00872-s001.pdf]

materials

Facile Tailoring of Contact Layer Characteristics of the Triboelectric Nanogenerator Based on Portable Imprinting Device

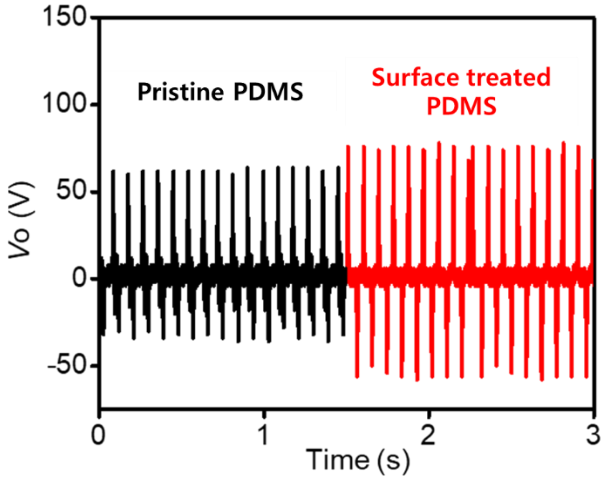

Figure S1. Output voltages generated from the TENG with the pristine PDMS contact layer and the ozone treated PDMS contact layer.
